# The Relationship Between Functional Decline of Care Recipients and Health of Caregivers

**DOI:** 10.1093/geroni/igaa057.1161

**Published:** 2020-12-16

**Authors:** Jinmyoung Cho, Alan Stevens

**Affiliations:** Baylor Scott & White Health, Temple, Texas, United States

## Abstract

Pearlin’s stress-process model (2010) depicts that functional decline in care-recipient would shape caregiving burden and impact on caregiver’s health. With this background, we explored how the changes in care-recipients’ physical and cognitive functioning are related to the caregivers’ physical health. A total of 853 care-recipients from the Rounds 1 and 5 of the National Health and Aging Trend Study (NHATS) and their 1,303 caregivers from Round 5 of National Study of Caregiving (NSOC) were included. Multiple regression analyses were conducted to identify correlates of self-rated health and the number of chronic conditions with the change in physical and cognitive functioning from Round 1 to 5 and multidimensional caregiver burden. Physical functioning measured by the NHATS short physical performance battery included balance stands, walking chair stands, grip strength, and peak airflow. Memory, orientation, and executive functioning measured cognitive functioning. Multidimensional caregiver burden includes four domains (emotional, psychological, relationship, and resilience) identified with factor analysis. Background factors (recipient’s age, assisting recipients for 5 years, race/ethnicity, and number of chronic conditions of recipient) were included as covariates. After controlling covariates, the data showed that 5-year change of physical functioning and caregiver’s emotional burden were negatively significant for self-rated health; and assisting care-recipients for 5 years or more was significant for more numbers of chronic conditions among caregivers. Findings highlight that caregivers’ physical health is closely associated with care-recipient’s functional decline for long-term caregiving experiences. Further investigation on caregiver’s physical health using multiple health outcomes is needed to promote physical health in long-term caregivers.

